# Hsa_circRNA_001676 accelerates the proliferation, migration and stemness in colorectal cancer through regulating miR-556-3p/G3BP2 axis

**DOI:** 10.1038/s41598-023-45164-6

**Published:** 2023-10-26

**Authors:** Qin Hao, Miao Zhang, Yingcai Wu, Yuchen Guo, Yanling Zheng, Lijuan Wu, Li Feng, Zhenfei Wang

**Affiliations:** 1 Department of Gastrointestinal Surgery, Affiliated Hospital of Inner Mongolia Medical University, Hohhot, 010051 China; 2 Graduate School, Inner Mongolia Medical University, Hohhot, 010010 China; 3 The Laboratory for Tumor Molecular Diagnosis, Peking University Cancer Hospital (Inner Mongolia Campus)/Affiliated Cancer Hospital of Inner Mongolia Medical University, Hohhot, 010020 China; 4 Department A of Abdominal surgery, Peking University Cancer Hospital (Inner Mongolia Campus)/Affiliated Cancer Hospital of Inner Mongolia Medical University, Hohhot, 010020 China

**Keywords:** Colorectal cancer, Colorectal cancer, Cancer metabolism, Colorectal cancer, Cell biology, Molecular biology

## Abstract

Circular RNAs (circRNAs) play key roles in colorectal cancer (CRC) progression, but little is known about the biological functions of hsa_circRNA_001676 in CRC. Therefore, we explored the potential role of hsa_circRNA_001676 in CRC development. RT-qPCR was performed to determine hsa_circRNA_001676, miR-556-3p and Ras-GTPase-activating SH3 domain-binding-proteins 2 (G3BP2) levels in CRC tissues. Meanwhile, to evaluate the roles of hsa_circRNA_001676, miR-556-3p and G3BP2 on CRC, functional analysis of cell proliferation, migration and stemness were then performed. Our results showed that compared to normal tissues, hsa_circRNA_001676 and G3BP2 level was elevated, but miR-556-3p level was reduced in CRC tissues. Additionally, luciferase reporter results showed that hsa_circRNA_001676 was shown to target miR-556-3p, and G3BP2 was targeted by miR-556-3p. Hsa_circRNA_001676 or G3BP2 overexpression promoted CRC cell proliferation and migration. Conversely, miR-556-3p overexpression suppressed CRC cell proliferation and migration. Moreover, deficiency of hsa_circRNA_001676 or G3BP2 repressed the CRC cell proliferation, migration and stemness. Meanwhile, hsa_circRNA_001676 deficiency obviously reduced tumor growth and stemness in a CRC mouse xenograft model. Furthermore, hsa_circRNA_001676 deficiency notably reduced G3BP2 level, but elevated miR-556-3p level in tumor tissues from tumor-bearing mice. Mechanistically, hsa_circRNA_001676 targeted miR-556-3p to increase G3BP2 level, contributing to the progression of CRC. Collectively, hsa_circRNA_001676 was able to accelerate proliferation, migration and stemness in CRC through regulating miR-556-3p/G3BP2 axis, suggesting that hsa_circRNA_001676 may become a potential therapeutic target in treating CRC.

## Introduction

Colorectal cancer (CRC) is a common malignancy in the digestive system, and is the second leading cause of cancer-related deaths worldwide^1^. Notwithstanding that adjuvant chemotherapy, chemoradiotherapy, immunotherapy and targeted therapy have been applied for treating CRC^2–4^, the prognosis remains worse in CRC patients^5,6^. Therefore, improving the diagnosis of CRC and developing new treatments for CRC patients are urgently needed.

Circular RNAs (circRNAs), a type of non-coding RNA molecules (ncRNAs), are formed by covalently closed loops with no 3′ poly(A) tails or 5′ end caps^7,8^. CircRNAs play crucial regulatory roles in different biological processes including cell growth and migration through sponging microRNA (miRNAs)^9^. MiRNAs are another class of ncRNAs with 17–22 nucleotides in length^10^. MiRNAs are able to repress gene expression through breaking translation or promoting mRNA degradation^11^. Mechanically, circRNAs can work as miRNA sponges, thereby repressing the ability of miRNAs to its target genes^12^.

CircRNAs have been shown to play key roles in cancer development, including CRC^13,14^. Some circRNAs are differentially expressed between normal and cancer tissues and may function as oncogenes or tumor suppressors^15^. For example, Circ3823 could accelerate CRC tumorigenesis, metastasis and angiogenesis via targeting miR-30c-5p/TCF7 axis^16^. CircEZH2 could aggravate CRC progression through stabilization of CREB1^17^. Meanwhile, circIFNGR2 notably facilitated CRC cell growth and increased cetuximab resistance through targeting miR-30b^18^. Conversely, hsa_circ_0001666 was able to restrain CRC progression through sponging miR-576-5p^19^. CircTMEM59 was found to target miR-668-3p/ID4 axis, thereby repressing CRC cell growth and metastasis^20^. In the present research, we identified that compared to normal tissues, hsa_circRNA_001676 was aberrantly upregulated in CRC tissues. Nevertheless, the role of hsa_circRNA_001676 in cancer progression remains largely unknown.

Furthermore, miR-556-3p was found to exert important roles in human cancers^21,22^. Additionally, G3BP2 protein was also found to be participated in the processes of tumor initiation and development^23,24^. However, the relationship among hsa_circRNA_001676, miR-556-3p and G3BP2 in CRC remains largely unknown. In this study, our results showed that miR-556-3p was targeted by hsa_circRNA_001676, and G3BP2 was targeted by miR-556-3p. Significantly, hsa_circRNA_001676 could affect CRC progression via targeting miR-556-3p/G3BP2 axis, suggesting that hsa_circRNA_001676 may become a potential therapeutic target in treating CRC.

## Materials and methods

### Clinical samples

A total of 3 pairs of CRC tissues and corresponding noncancerous tissues were obtained from the Peking University Cancer Hospital (Inner Mongolia Campus)/Affiliated Cancer Hospital of Inner Mongolia Medical University, conformed to the Declaration of Helsinki. Informed consent was got from all participants. Meanwhile, the Ethics Committee of Peking University Cancer Hospital (Inner Mongolia Campus)/Affiliated Cancer Hospital of Inner Mongolia Medical University approved this study.

### Bioinformatics analysis

The CRC-related circRNA expression profiles were downloaded from the Gene Expression Omnibus (GEO) database (GSE142837). The differentially expressed circRNAs (DEcircRNAs) between CRC tissues and corresponding noncancerous tissues were screened by using the R language and the GEO2R tool. Threshold criteria were: |log (Fold Change (FC)) |> 1 and -log10 (*P*-value) > 1.3.

### Cell culture

The normal colonic mucosa cell line NCM460 (GuangZhou Jennio Biotech Co., Ltd), HT29 and HCT116 cells (Procell), SW620 , SW480, LOVO and 293 T cells (Shanghai Cell Bank) were maintained in Dulbecco’s modified Eagle medium (DMEM) supplied with 10% fetal bovine serum (FBS) with 5% CO_2_.

### Cell transfection

The sequences of mimics or inhibitor negative control (NC), miR-556-3p mimics or inhibitor were synthesized by HANBIO. The pcDNA3.1-NC and pcDNA3.1-G3BP2 plasmids and siRNA (si) specifically targeting G3BP2 (si-G3BP2) and siRNA NC (si-NC) were constructed by HANBIO. HT29 cells were transfected with mimics NC, miR-556-3p mimics, si-NC or si-G3BP2; SW480 cells were transfected with inhibitor NC, miR-556-3p inhibitor, pcDNA3.1-NC or pcDNA3.1-G3BP2 using the lipofectamine 2000, respectively.

The hsa_circRNA_001676-overexpressing lentivirus (hsa_circRNA_001676-OE), short hairpin RNAs targeting hsa_circRNA_001676 (sh-hsa_circRNA_001676) were obtained from HANBIO. HT29 cells were infected with sh-hsa_circRNA_001676 and SW480 cells were infected with hsa_circRNA_001676-OE for 48 h respectively, and then stable-expressing CRC cells were chose by puromycin.

### Reverse transcription-quantitative PCR (RT-qPCR) assay

Total RNA from cells or tumor tissues were extracted using the Redzol reagent (SBS Genetech Co.,Ltd.). Next, the cDNA was synthesized using the Surescript™ First-Strand cDNA Synthesis Kit (iGeneBio). After that, qPCR was conducted with the SYBR Green qPCR Master Mix (None ROX) kit (Servicebio). U6 was used as an internal control for miR-556-3p, and GAPDH was used as an internal control for hsa_circRNA_001676, G3BP2, OCT4 and Nanog. The 2^−ΔΔCt^ method was used for determining relative gene levels.

### Cell counting Kit-8 (CCK-8) assay

HT29 and SW480 cells were seeded onto 96-well plates overnight at 37 °C. After indicated treatment, each well was added with CCK-8 reagent (10 μl, Beyotime). Next, after another 4 h of incubation at 37 °C, the optical density (OD) was measured under a micro-plate reader (DNM-9602; PERLONG), the wavelength was set to 490 nm.

### Colony formation assay

HT29 and SW480 cells were loaded onto 12-well plates overnight at 37 °C. After indicated treatment, cells were then incubated for 1 week at 37 °C. Thereafter, cells were stained with 0.1% crystal violet solution for 30 min. Finally, the colonies were captured under a light microscope.

### Wound healing assay

HT29 and SW480 cells were plated into 12-well plates overnight at 37 °C. The wounds were then created by using a sterilized pipet tip. After 0, 24 or 48 h of incubation, images were taken using a light microscope.

### Transwell assay

Cell migration ability was assessed using a 24-well transwell chamber (Corning) with an 8 µm-pore size filter membrane. Cells suspended in DMEM without serum (200 µl) were loaded onto the upper Transwell cell inserts, and DMEM medium (500 µl) with 10% FBS was seeded onto the bottom chambers. After 24 h of incubation at 37 °C, crystal violet solution (0.1%) was used for staining the cells on the lower surface of the filter. Finally, the migrated cells were observed under a light microscope and counted in three randomly selected regions.

### Spheroid formation assay

Cancer stemness was assessed by the spheroid formation assay. Cells were suspended in serum-free DMEM containing 2% B27 + 20 ng/ml epidermal growth factor (EGF) + 10 ng/ml of basic fibroblast growth factor (bFGF)^25^, and then added into a 6-well ultralow attachment plate. After 2 weeks of incubation at 37 °C, the spheres were photographed and counted under a light microscope. A Spheroid containing > 50 cells were counted.

### Dual-Luciferase Reporter Assay

The wild-type (wt) or mutant (mut) 3′-untranslated regions (UTRs) of hsa_circRNA_001676 and G3BP2 were cloned into the pmirGLO luciferase reporter plasmids. Next, these plasmids and miR-556-3p mimics or inhibitor were co-transfected into 293 T cells using the lipofectamine 2000, respectively. Subsequently, the luciferase activity was assessed by a Dual Luciferase Reporter Assay kit (Beyotime) at 48 h.

### Western blot assay

Protein samples were separated with 10% SDS-PAGE gels and followed by transferring onto a PVDF membrane. Later on, the membrane was probed with primary antibodies at 4 °C overnight including anti-Oct-4 (No. ab109250), anti-Nanog (No. ab205481), anti-G3BP2 (No. ab190011), anti-CD133 (No. ab19898), anti-CD44 (No. ab189524) and anti-GAPDH (No. Ab9485, Abcam) antibodies, followed by incubation with a HRP-conjugated secondary antibody (No. Ab6721, Abcam) at room temperature. Finally, blots were visualized using a LightningTM Chemiluminescence Reagent (PerkinElmer).

### TUNEL staining assay

Paraffin-embedded tumor tissues were cut into 4-μm thick slices. Next, the TUNEL Apoptosis Detection Kit (FITC) purchased from BOSTER was used for detecting cell apoptosis in tumor tissues according to the manufacturer's instructions. The nuclei was counterstained with DAPI (Solarbio). Images were then observed under a fluorescence microscope (OLYMPUS).

### Immunohistochemical (IHC) and immunofluorescence (IF) staining assays

For IHC assay, sections were blocked in normal goal serum at room temperature for 20 min and then probed with anti-G3BP2 antibody (No. ab190011), anti-Oct-4 antibody (No. ab200834), anti-Nanog antibody (No. ab109250, Abcam) overnight at 4 °C. Next, sections were stained with HRP-labeled secondary antibody (No. Ab6721) for 20 min at room temperature. After visualizing with DAB solution, photographs were taken with a light microscope (OLYMPUS).

For IF assay, sections were probed with anti-CD133 antibody (No. ab19898, Abcam) and anti-CD44 antibody (No. ab238464, Abcam) overnight at 4 °C and then probed with the fluorescently-labeled secondary antibody (No. ab150077). Finally, images were taken with a fluorescence microscope (OLYMPUS). The Image Pro Plus software was used for semiquantitative analysis.

### Animal study

All animal experiments were approved by the Animal Care and Use Committee of the Peking University Cancer Hospital (Inner Mongolia Campus)/Affiliated Cancer Hospital of Inner Mongolia Medical University, and conducted in accordance with the National Institutes of Health Guidelines for Care and Use of Laboratory Animals and carried out in accordance with the ARRIVE guidelines. A total of 20 BALB/c nude mice (18–20 g, Vital River) were grouped into three groups randomly: Control, NC and sh-hsa_circRNA_001676 groups. Each mouse was injected subcutaneously with untransfected (control group; 2 × 10^6^ cells) or transfected HT29 cells (NC group and sh-hsa_circRNA_001676 group; 2 × 10^6^ cells). Tumor growth was monitored at indicated times. The tumor volume (V) was calculated as: tumor length × tumor width^2^/2. After 4 weeks, the mice were sacrificed by cervical dislocation under anesthesia (1% isoflurane inhalation), and tumors were dissected out from the sacrificed mice.

### Hematoxylin and eosin (H&E) staining assay

Paraffin-embedded tumor tissues were cut into 4-μm thick slices. After dewaxing and rehydration, sections were then subjected to H&E staining. Finally, images were observed under a light microscope.

### Statistical analysis

Each experiment was independently repeated at least three times. GraphPad Prism 8 software was used for statistical analysis. The differences between two groups were evaluating using an unpaired Student t‐test. Meanwhile, one‐way analysis of variance (ANOVA) was used for multiple comparison. Data are shown as mean ± standard deviation. *P* < 0.05 indicates statistical significance.

### Ethical approval and consent to participate

Informed consent was got from all participants and the Ethics Committee of Peking University Cancer Hospital (Inner Mongolia Campus)/Affiliated Cancer Hospital of Inner Mongolia Medical University approved this study.

All animal experiments were approved by the Animal Care and Use Committee of the Peking University Cancer Hospital (Inner Mongolia Campus)/Affiliated Cancer Hospital of Inner Mongolia Medical University, and conducted in accordance with the National Institutes of Health Guidelines for Care and Use of Laboratory Animals and carried out in accordance with the ARRIVE guidelines.

## Results

### Hsa_circRNA_001676 level was elevated in CRC tissues

**Figure 1:**
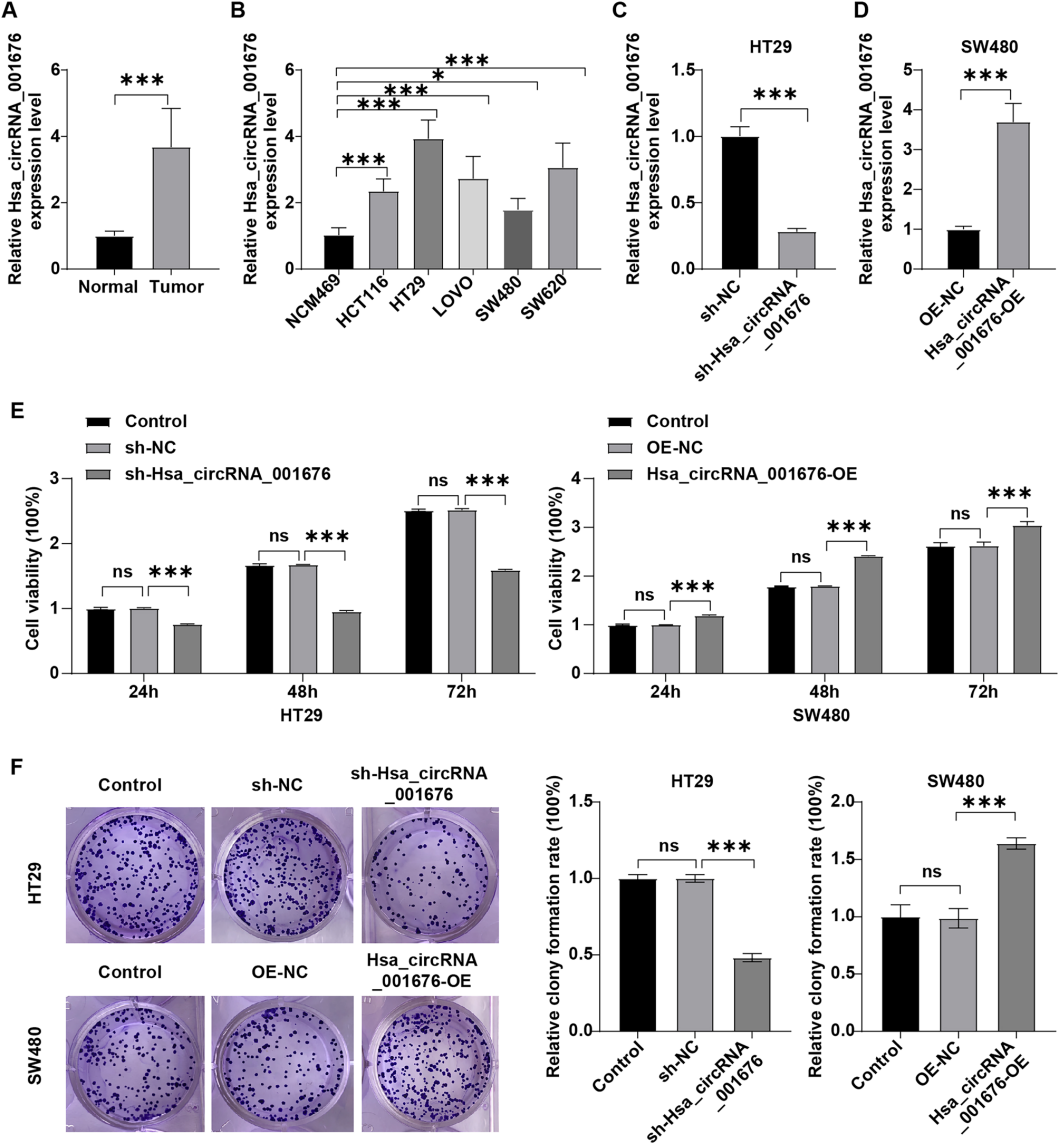
Hsa_circRNA_001676 enhanced CRC cell viability. (**A**) RT-qPCR analysis of hsa_circRNA_001676 level in CRC tissues (n = 3) and paired noncancerous tissues (n = 3). (**B**) RT-qPCR analysis of hsa_circRNA_001676 level in NCM460 cells and different CRC cells. (**C**) RT-qPCR analysis of hsa_circRNA_001676 level in HT29 cells transfected with sh-NC or sh-hsa_circRNA_001676. (**D**) RT-qPCR analysis of hsa_circRNA_001676 level in SW480 cells transfected with OE-NC or hsa_circRNA_001676-OE. (**E, F**) HT29 cells were transfected with sh-NC or sh-hsa_circRNA_001676 and SW480 cells were transfected with OE-NC or hsa_circRNA_001676-OE. (**E**) CCK-8 and (**F**) colony formation assays were applied for evaluating cell viability and proliferation, respectively. *P < 0.05; **P < 0.01; ***P < 0.001.

By comparing circRNA levels in five pairs of noncancerous tissues and CRC tissues from CRC patients, a circRNA expression profile was downloaded from the GSE142837 dataset. The volcano plot disclosed that compared to the matched normal tissues, hsa_circRNA_001676 level was obviously elevated in CRC samples (Fig. S1), which was verified by the RT-qPCR results in Fig. 1A. Moreover, compared to NCM460 cells, higher level of hsa_circRNA_001676 was detected in CRC cells (Fig. 1B). Meanwhile, among these five CRC cells, hsa_circRNA_001676 was expressed at the highest level in HT29 cells and expressed at the lowest level in SW480 cells (Fig. 1B). Thus, we reduced hsa_circRNA_001676 level in HT29 cells by using sh-Hsa_circRNA_001676, and overexpressed hsa_circRNA_001676 level in SW480 cells by using Hsa_circRNA_001676-OE (Fig. 1C and D). Significantly, hsa_circRNA_001676 expression was declined in HT29 cells transfected with sh-hsa_circRNA_001676 plasmids and increased in SW480 cells transfected with hsa_circRNA_001676-OE plasmids (Fig. 1C and D).

### Hsa_circRNA_001676 enhanced CRC cell proliferation, migration and stemness

**Figure 2:**
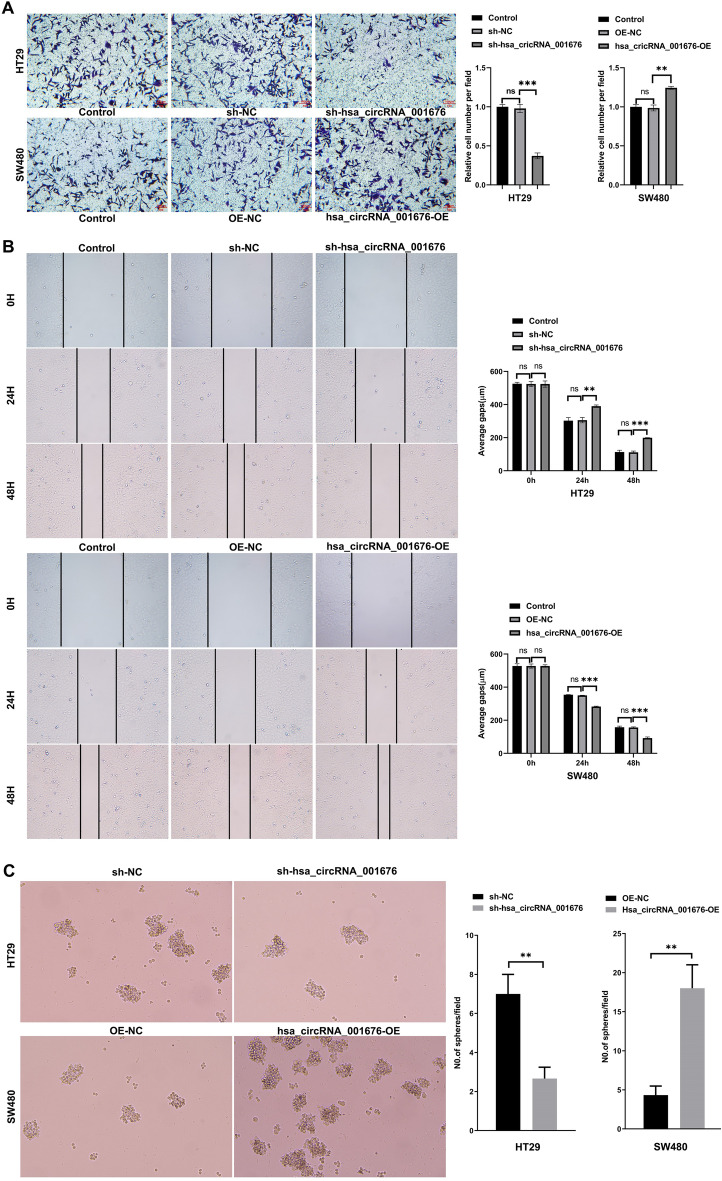
Hsa_circRNA_001676 enhanced CRC cell migration and stemness. HT29 cells were transfected with sh-NC or sh-hsa_circRNA_001676 and SW480 cells were transfected with OE-NC or hsa_circRNA_001676-OE. (**A**) Transwell and (**B**) wound healing assays were performed to assess cell migration (Scale bar = 100 μm). (**C**) Sphere formation efficiency was determined by using the sphere formation assay. **P < 0.05; ***P < 0.01.

To explore the role of hsa_circRNA_001676 in CRC, CCK-8 and colony formation assays were conducted. Obviously, silenced hsa_circRNA_001676 strongly repressed HT29 cell viability and proliferation, whereas forced expression of hsa_circRNA_001676 enhanced SW480 cell viability and proliferation (Fig. 1E and F). Additionally, the results of transwell, wound healing and spheroid formation assays indicated that silenced hsa_circRNA_001676 weakened HT29 cell migration and sphere formation abilities, whereas hsa_circRNA_001676 overexpression displayed the opposite effects on SW480 cells (Fig. 2A–C). Collectively, hsa_circRNA_001676 could accelerate CRC cell growth, migration and stemness.

### Hsa_circRNA_001676 functioned as a sponge of miR-556-3p

**Figure 3:**
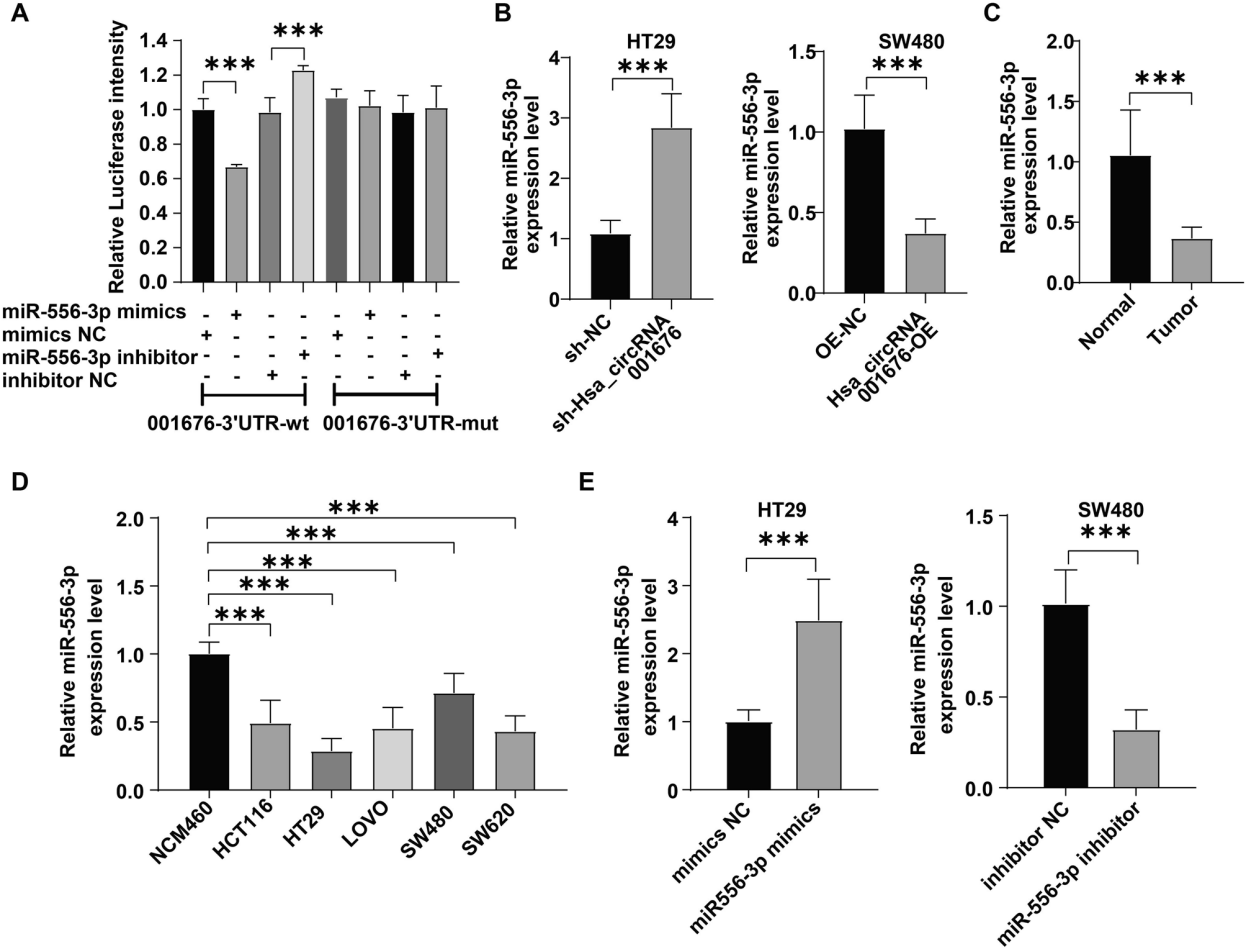
Hsa_circRNA_001676 functioned as a sponge of miR-556-3p. (**A**) The luciferase activities were measured in 293 T cells co-transfected with hsa_circRNA_001676 3’UTR-wt or -mut and miR-556-3p mimics or inhibitor. (**B**) RT-qPCR analysis of miR-556-3p level in HT29 or SW480 cells transfected with sh-hsa_circRNA_001676 or hsa_circRNA_001676-OE, respectively. (**C**) RT-qPCR analysis of miR-556-3p level in CRC tissues (n = 3) and paired noncancerous tissues (n = 3). (**D**) RT-qPCR analysis of miR-556-3p level in NCM460 cells and different CRC cells. (**E**) RT-qPCR analysis of miR-556-3p level in HT29 and SW480 cells transfected with miR-556-3p mimics or inhibitor, respectively. ***P < 0.001.

Previous studies have shown that circRNAs could function as miRNA “sponges” to “adsorb” miRNAs^26,27^. This process greatly eliminated the inhibitory effects of miRNAs on target genes^27^. Therefore, to identify the downstream miRNAs of hsa_circRNA_001676, Circbank database (http://www.circbank.cn/) and CircInteractome database (https://circinteractome.irp.nia.nih.gov/) were used. The results of dual-luciferase reporter assay showed that the luciferase activity in wt-hsa_circRNA_001676-transfected 293 T cells was declined by miR-556-3p mimics and elevated by miR-556-3p inhibitor (Fig. 3A). Moreover, sh-hsa_circRNA_001676 greatly elevated miR-556-3p level in HT29 cells, whereas hsa_circRNA_001676-OE remarkably declined miR-556-3p level in SW480 cells (Fig. 3B). These results verified the interaction between miR-556-3p and hsa_circRNA_001676 in CRC.

**Figure 4:**
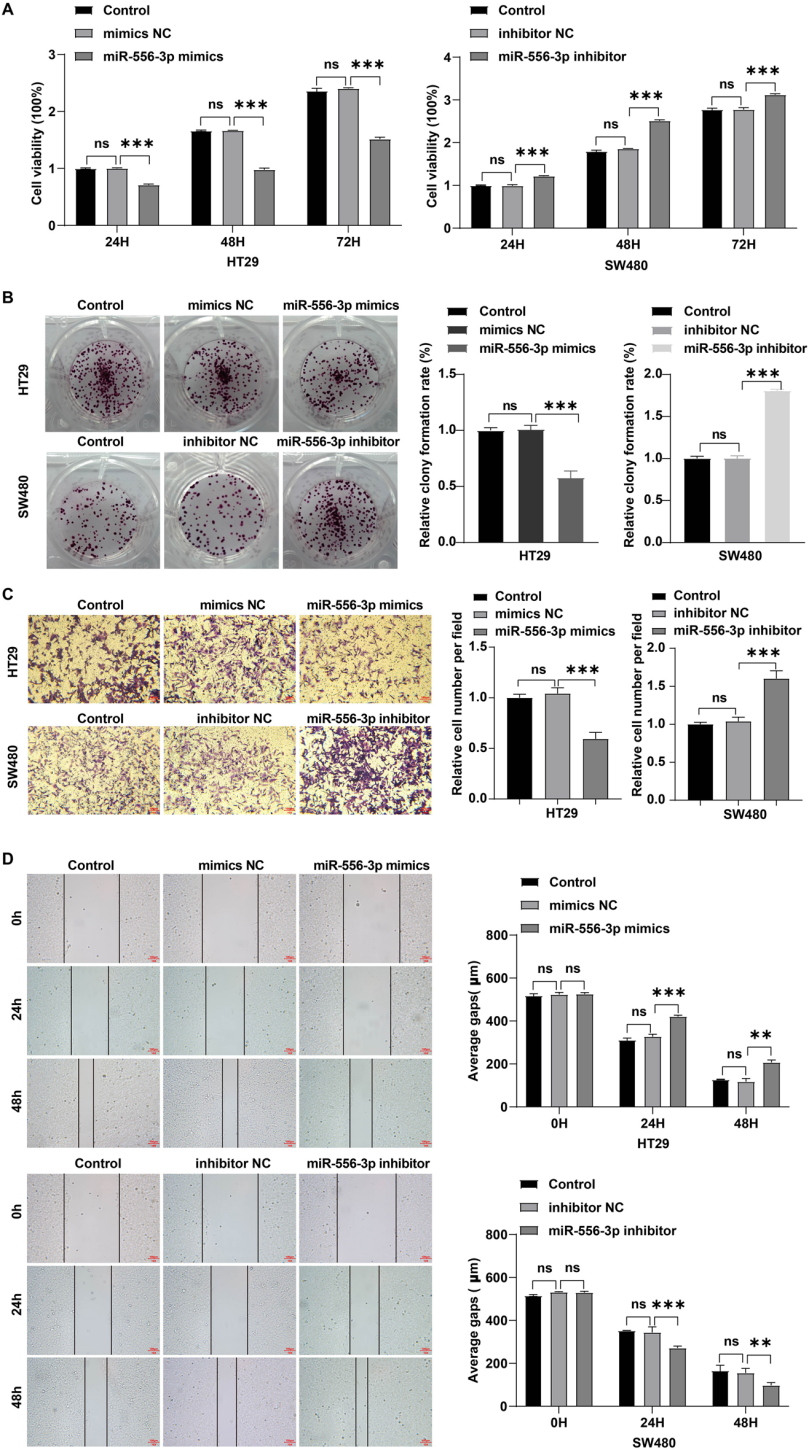
MiR-556-3p weakened the proliferation and migration of CRC cells. SW480 cells were transfected with miR-556-3p inhibitor and HT29 cells were transfected with miR-556-3p mimics. (**A**) CCK-8 and (**B**) colony formation assays were applied for evaluating cell viability and proliferation, respectively. (**C**) transwell and (**D**) wound healing assays were performed to assess cell migration (Scale bar = 100 μm). *P < 0.05; **P < 0.01; ***P < 0.001.

Additionally, compared to normal noncancerous tissues and normal colonic mucosa cells, CRC tissues and CRC cells evidenced a lower miR-556-3p level respectively (Fig. 3C and D). MiR-556-3p mimics dramatically elevated miR-556-3p level in HT29 cells, whereas miR-556-3p inhibitor greatly reduced miR-556-3p level in SW480 cells (Fig. 3E). Moreover, miR-556-3p mimics markedly repressed HT29 cell viability, proliferation and migration; however, miR-556-3p inhibitor displayed the opposite effects on SW480 cells (Fig. 4A–D).

### G3BP2 is a downstream binding target of miR-556-3p

**Figure 5:**
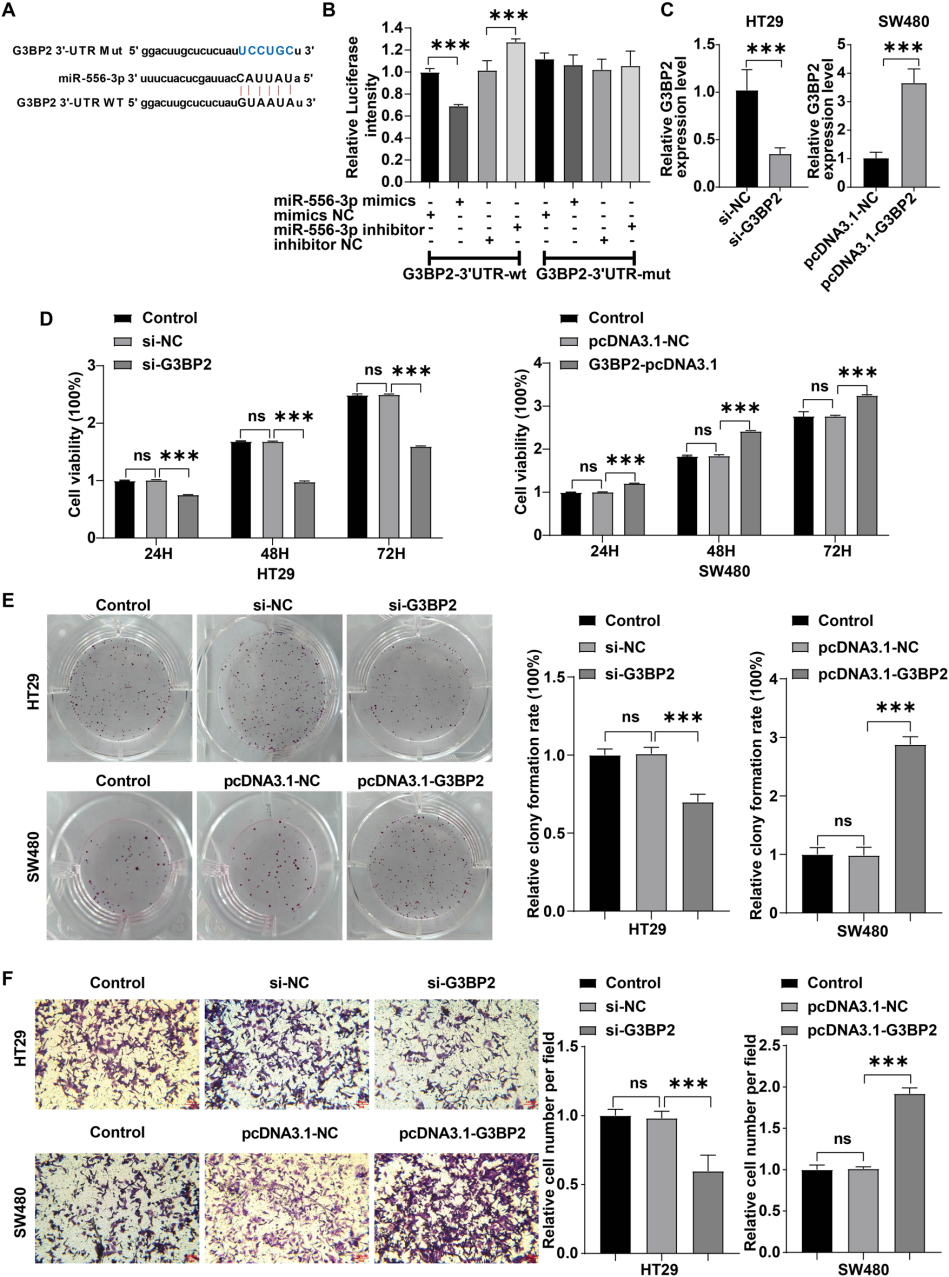
G3BP2 was a downtream binding target of miR-556-3p. (**A**) The binding sites between G3BP2 and miR-556-3p was obtained from Starbase databases. (**B**) The luciferase activities were measured in 293 T cells co-transfected with G3BP2 3’UTR-wt or -mut and miR-556-3p mimics or inhibitor. (**C**) HT29 cells were transfected with si-G3BP2 and SW480 cells were transfected with pcDNA3.1-G3BP2. RT-qPCR was conducted to determine G3BP2 level in CRC cells. (**D**, **E**) CCK-8 and colony formation assays were applied for evaluating cell viability and proliferation, respectively. (**F**) The transwell assay were performed to assess cell migration (Scale bar = 100 μm). **P < 0.05; ***P < 0.01; ****P < 0.001.

Next, Starbase (https://starbase.sysu.edu.cn/) database predicted the downstream targets of miR-556-3p. Over 3000 target genes were listed in Starbase. Among these, G3BP2 could regulate cancer stemness via upregulating the expressions of stem cell markers Oct-4 and Nanog^23^. Our results found that compared to normal controls, cell apoptosis was attenuated in CRC tissues (Fig. S2A). Meanwhile, the expressions of Oct-4, Nanog, CD133, CD44 and G3BP2 were obviously elevated in CRC tissues (Figs. S2B–E), suggesting that these tumor tissues displayed high self-renewal capability and high metastatic potential^28^. Meanwhile, the effect of G3BP2 on stemness in CRC and the relationship between G3BP2 and hsa_circRNA_001676/miR-556-3p in CRC remain elusive. Thus, we focused on G3BP2 because of its role in cancer stemness in the study. The data in Starbase showed that 3’UTR of G3BP2 was complementary to miR-556-3p sequences (Fig. 5A). Additionally, miR-556-3p mimics notably repressed the luciferase activity in wt-G3BP2 group cells (Fig. 5B), suggesting G3BP2 could be a direct target of miR-556-3p.

Furthermore, silenced G3BP2 greatly declined G3BP2 level in HT29 cells, as well as repressed HT29 cell viability, proliferation and migration; conversely, G3BP2 overexpression strongly elevated G3BP2 level in SW480 cells, as well as facilitated SW480 cell viability, proliferation and migration (Figs. 5C-F and S3A-3B). Collectively, G3BP2 could act as an oncogene in CRC cells.

### G3BP2 promoted CRC cell stemness

**Figure 6:**
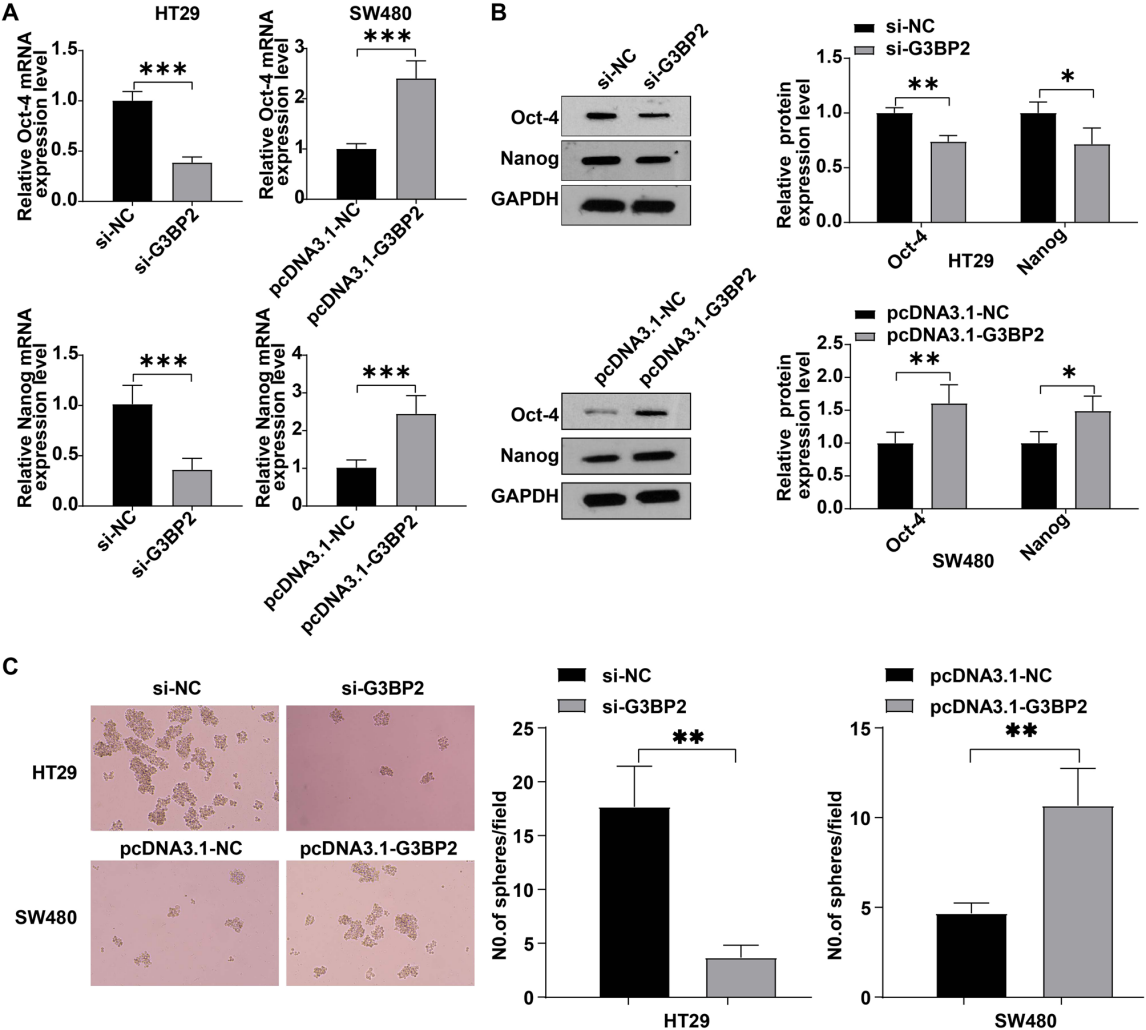
G3BP2 promoted CRC cell stemness. HT29 cells were transfected with si-G3BP2 and SW480 cells were transfected with pcDNA3.1-G3BP2. (**A**) RT-qPCR and (**B**) western blot analysis of Oct-4 and Nanog level in transfected cells. (**C**) Sphere formation efficiency was determined by using the sphere formation assay. *P < 0.05; **P < 0.01; ***P < 0.001.

Next, we tested the effect of G3BP2 on CRC cell stemness in vitro*.* As revealed in Fig. 6A and B, Oct-4 and Nanog levels were declined in CRC cells by silencing of G3BP2, and increased by overexpression of G3BP2. Additionally, G3BP2 deficiency dramatically weakened the sphere formation ability of HT29 cells, whereas G3BP2 overexpression notably enhanced the sphere-forming capacity of SW480 cells (Fig. 6C). To sum up, G3BP2 could promote CRC cell stemness.

### Hsa_circRNA_001676 deficiency reduced tumor growth and stemness in CRC in vivo by regulating miR-556-3p/G3BP2 axis

**Figure 7:**
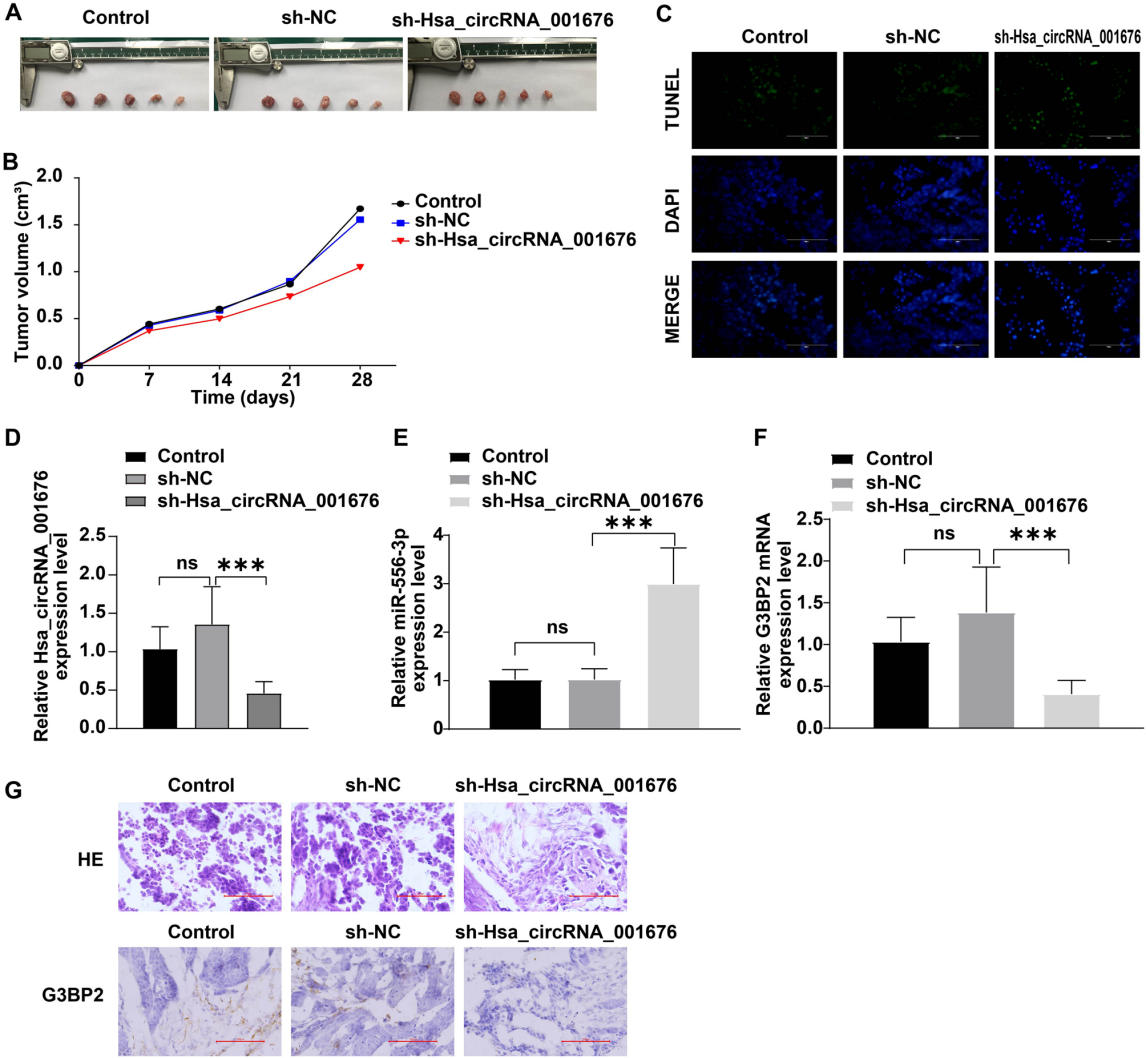
Hsa_circRNA_001676 deficiency reduced tumor growth of CRC in vivo by regulating miR-556-3p/G3BP2 axis. (**A**) Photographs of xenograft tumors. (**B**) Curves of tumor volumes. (**C**) TUNEL staining analysis of cell apoptosis in tumor tissues (Scale bar = 100 μm). RT-qPCR analysis of (**D**) hsa_circRNA_001676, (**E**) miR-556-3p and (**F**) G3BP2 levels in tumor tissues. (**G**) H&E (upper panel) stained histological images of the tumor tissues. IHC (lower panel) analysis of G3BP2 protein expression in tumor tissues (Scale bar = 100 μm). *P < 0.05; ***P < 0.001.

**Figure 8:**
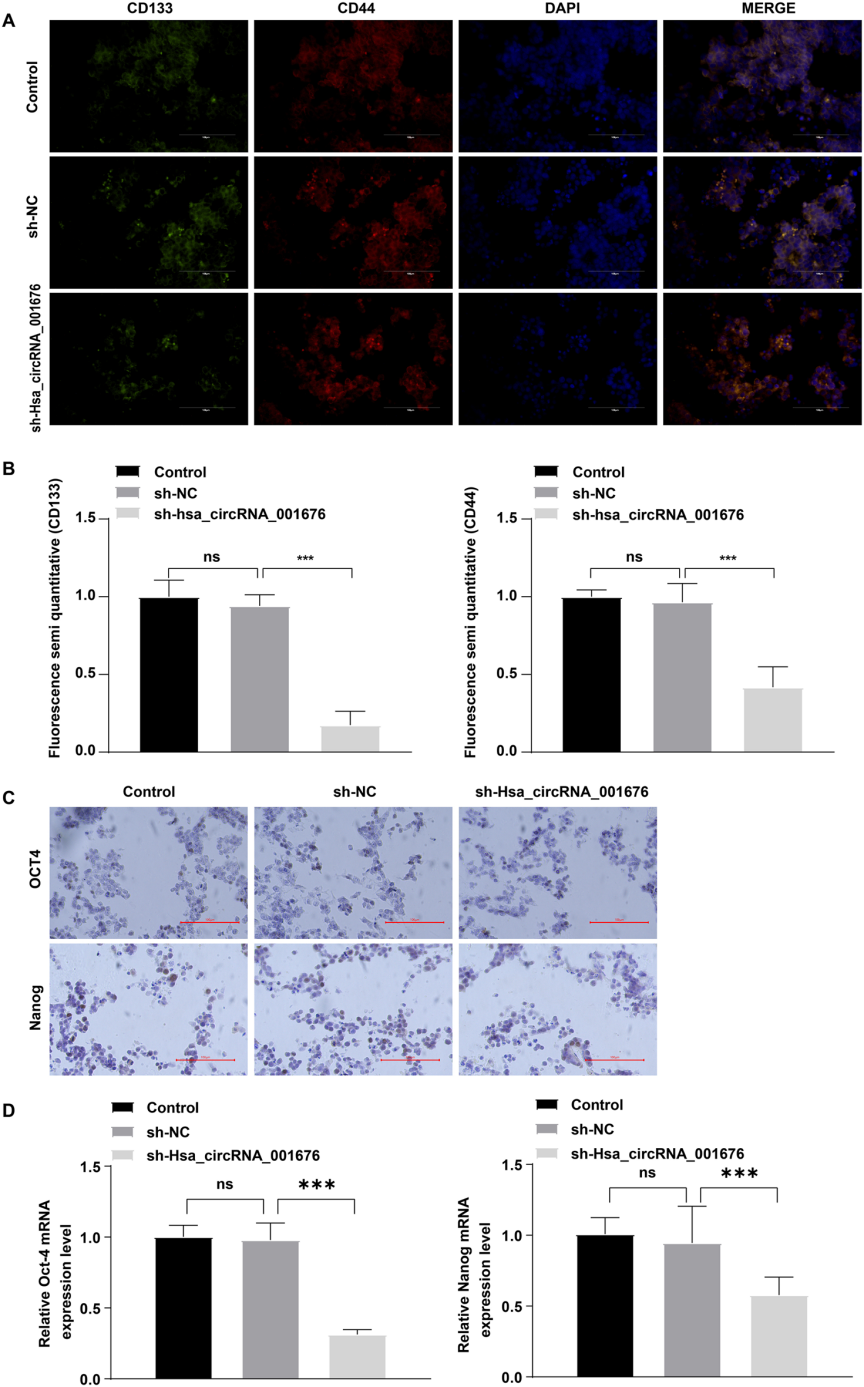
Hsa_circRNA_001676 deficiency reduced the stemness of CRC in vivo by regulating miR-556-3p/G3BP2 axis. (**A**, **B**) IF analysis of CD133 and CD44 protein expressions in tumor tissues. (**C**) IHC and (**D**) RT-qPCR analysis of Oct-4 and Nanog levels in tumor tissues. Scale bar = 100 μm. **P < 0.01; ***P < 0.001.

A xenograft mouse model with CRC was constructed to further explore the role of hsa_circRNA_001676 in CRC. As shown in Fig. 7A and B, hsa_circRNA_001676 deficiency obviously reduced the tumor volume and triggered tumor cell apoptosis (Fig. 7A–C). Meanwhile, hsa_circRNA_001676 downregulation remarkably declined hsa_circRNA_001676 and G3BP2 levels, and elevated miR-556 level in tumor tissues (Fig. 7D–G). Furthermore, compared to the sh-NC group, downregulation of hsa_circRNA_001676 led to significant decreases in CD133, CD44, Oct-4 and Nanog levels in tumor tissues (Fig. 8A–D). Collectively, hsa_circRNA_001676 deficiency was able to hamper CRC progression in vivo by regulating miR-556-3p/G3BP2 axis.

## Discussion

CircRNAs have been shown to exhibit a regulatory role in CRC progression^29^. However, the biological role of a large number of circRNAs in CRC progression remain not well clarified. In this study, we found that hsa_circRNA_001676 level was elevated in CRC tissues and CRC cell lines. Additionally, downregulation of hsa_circRNA_001676 could suppress tumor growth and stemness in CRC in vitro and in vivo. Conversely, hsa_circRNA_001676 overexpression strongly accelerated tumor growth and stemness in CRC in vitro. Our results showed that hsa_circRNA_001676 could function as a new oncogenic driver to facilitate tumor growth and stemness in CRC.

Cancer stemness has been recognized as the leading cause of cancer metastasis and relapse^30^. Evidence has shown that some cancer cells possess typical stemness properties, which are important for the initiation and metastasis of tumors^31^. Targeting cancer stemness is a promising approach to fight CRC^32^. CircRNAs play a key role in modulating cancer stemness^33,34^. For example, CircPTN enhanced glioma cell stemness and self-renewal via sponging miR-145-5p^35^. CircAGFG1 could facilitate the stemness and metastasis in CRC through targeting miR-4262 and miR-185-5p^36^. Circ_0030586 retarded bladder cancer cell growth and stemness through targeting miR-665^37^. Our data showed that deficiency of hsa_circRNA_001676 remarkably repressed the sphere-formation ability of CRC cells in vitro and reduced CD133, CD44, Oct-4 and Nanog levels in tumor tissues in vivo, suggesting that deficiency of hsa_circRNA_001676 could suppress CRC cell stemness. However, the mechanism by which hsa_circRNA_001676 affects the stemness in CRC remains unknown.

CircRNAs have been reported to exert their regulatory roles in cancer development via functioning as miRNA “sponges”^38^. In this study, Circbank and CircInteractome databases were applied to predict the targets of hsa_circRNA_001676. The data showed that hsa_circRNA_001676 could interact with miR-556-3p. Li et al. found that inhibition of miR-556-3p could enhance gastric cancer cell growth^21^. Conversely, diminished miR-556-3p repressed hemangioma cell proliferation through upregulating VEGFC^22^. These findings demonstrated that miR-556-3p may be expressed both as a tumor suppressor or an oncogene in different cancers. Our results showed that compared to normal colonic mucosa cells, miR-556-3p level was remarkably reduced in CRC cells. Forced miR-556-3p expression greatly repressed CRC cell proliferation and migration, suggesting that miR-556-3p might tumor suppressor gene in CRC.

Furthermore, in this study, miR-556-3p was shown to target G3BP2. It has been shown that G3BP2 often overexpressed in some cancers and acted as an oncogene^39,40^. Overexpression of G3BP2 could expedite ESCC cell migration and invasion^39^. Inhibition of G3BP2 could decline PD-L1 expression in cancer cells, thereby facilitating anticancer immunotherapy^40^. Consistent with previous studies, we verified that G3BP2 overexpression could enhance CRC cell proliferation and migration, indicating that G3BP2 could act as an oncogene in CRC. Additionally, G3BP2 could trigger tumor initiation in breast cancer via upregulation of Oct-4 and Nanog^23^. Meanwhile, Oct-4 and Nanog are important stemness-associated mediators in the maintenance of cancer stemness^41,42^. Reducing Oct-4 and Nanog levels in CRC cells could repress the cancer stemness properties^43^. Moreover, high Oct-4 and Nanog levels are related to worse prognosis in CRC^44^. These findings above suggested a relationship between G3BP2 and cancer stemness. For the first time, we found that G3BP2 overexpression could upregulate Oct-4 and Nanog levels in CRC cells, suggesting that G3BP2 could facilitate CRC cell stemness.

Furthermore, circHERC4 could elevate E-cadherin protein level in CRC through inactivating miR-556-5p, thereby facilitating tumor migration and metastasis^45^. Circ_0020378 could facilitate osteosarcoma cell migration and growth via sponging miR-556-5p^46^. Meanwhile, circ-ABCB10 could affect cisplatin sensibility in lung cancer through binding with miR-556-3p^47^. Moreover, circFNDC3B could repress oncogene G3BP2 via binding with miR-1178-3p, thus suppressing bladder cancer progression^48^. These findings showed the relationships between different circRNAs and miR-556 or G3BP2. In this research, forced hsa_circRNA_001676 expression notably reduced miR-556-3p level, and elevated G3BP2, Oct-4 and Nanog levels in tumor tissues. These results showed that hsa_circRNA_001676 could act as a miR-556-3p sponge to weaken the inhibitory effects of miR-556-3p on its target G3BP2, thereby upregulating Oct-4 and Nanog levels.

It has been shown that one miRNA can target multiple genes and one circRNA also can sponge various miRNAs^49,50^. In this study, we only illustrated that hsa_circRNA_001676 could affect CRC cell growth and stemness through miR-556-3p/G3BP2 axis. However, further study is needed to uncover more hsa_circRNA_001676-associated miRNAs or mRNAs, and this research might further illustrate the molecular mechanism of hsa_circRNA_001676 in CRC.

## Conclusions

This study is the first to research hsa_circRNA_001676/miR-556-3p/G3BP2 axis in CRC. In summary, Hsa_circRNA_001676 was able to accelerate proliferation, migration and stemness in CRC through targeting miR-556-3p/G3BP2 axis, demonstrating that hsa_circRNA_001676 may serve as a potential target for treating CRC.

### Supplementary Information

Supplementary Information 1.

Supplementary Information 2.

## Data Availability

The data supporting the conclusions of this study are included in this article.
