# Supplementary figures and images for: Hsa_circRNA_001676 accelerates the proliferation, migration and stemness in colorectal cancer through regulating miR-556-3p/G3BP2 axis
Source: Sci Rep. 2023 Oct 26;13:18353. doi: 10.1038/s41598-023-45164-6 (PMC10603078; doi:10.1038/s41598-023-45164-6)

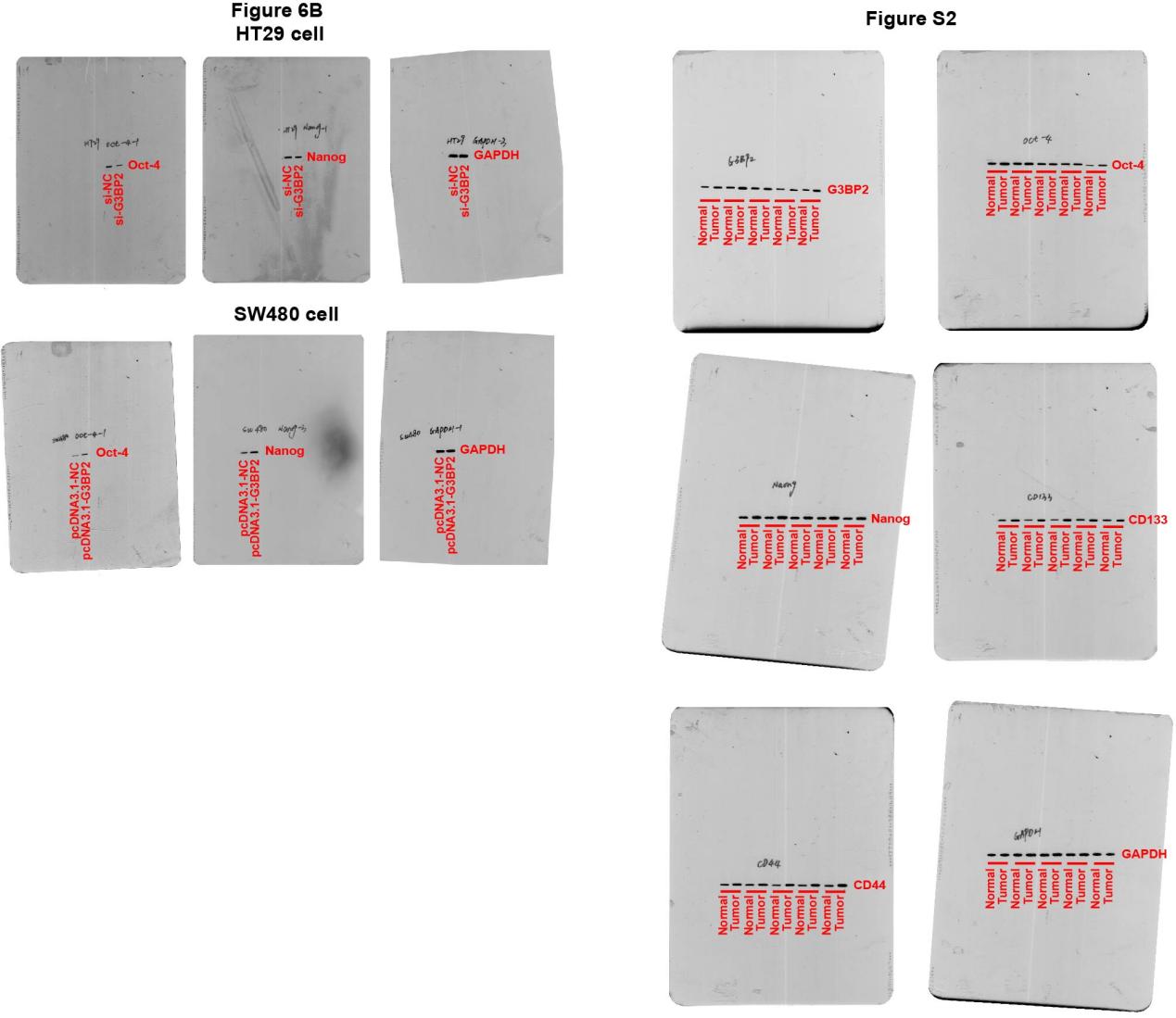

Supplement: Supplementary file 1 — Supplementary Information 1. [file 41598_2023_45164_MOESM1_ESM.docx]
