# Supplementary material for: Hsa_circRNA_001676 accelerates the proliferation, migration and stemness in colorectal cancer through regulating miR-556-3p/G3BP2 axis
Source: Sci Rep. 2023 Oct 26;13:18353. doi: 10.1038/s41598-023-45164-6 (PMC10603078; doi:10.1038/s41598-023-45164-6)
Supplement: Supplementary file 2 — Supplementary Information 2. [file 41598_2023_45164_MOESM2_ESM.docx]

**
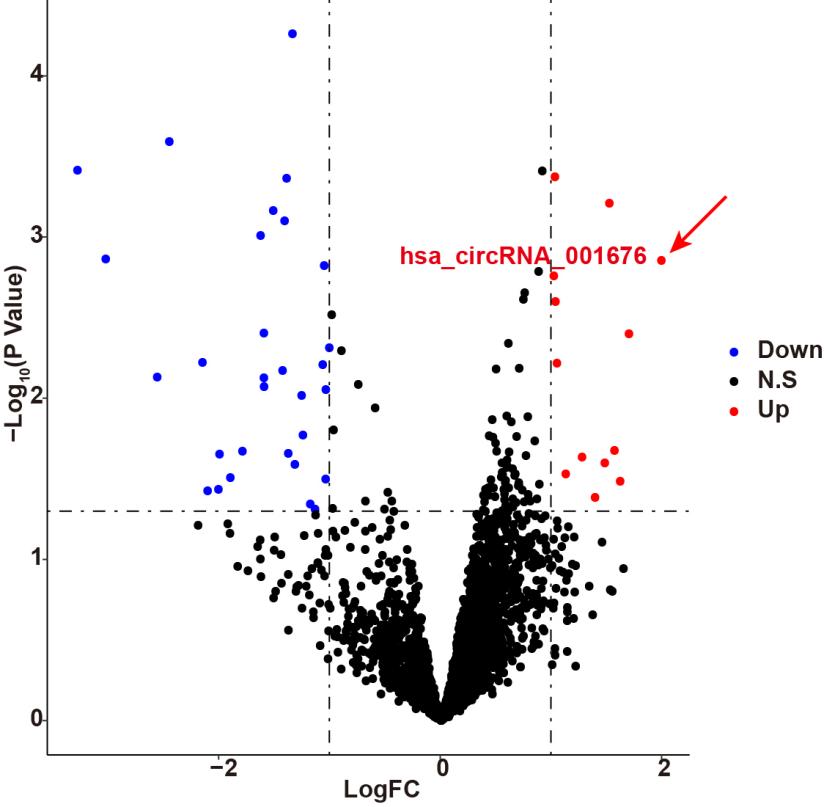
**

**Figure S1 Hsa_circRNA_001676 exhibited upregulation in CRC. V**olcano plot showed the DEcircRNAs bewteen CRC tissues and corresponding noncancerous tissues in the GSE142837 dataset.

**
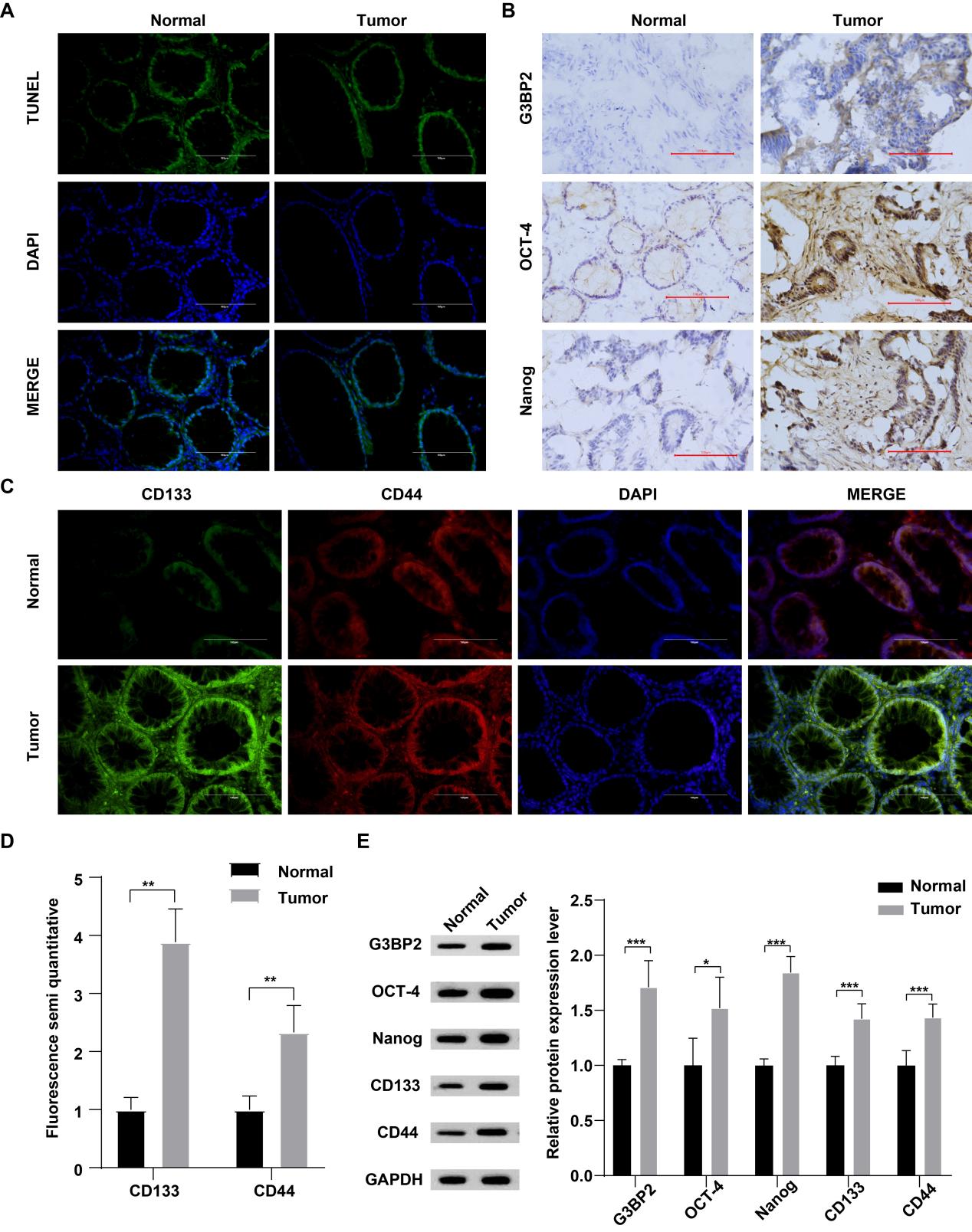
Figure S2 G3BP2 was increased in CRC tissues. (A)** TUNEL staining analysis of cell apoptosis in CRC tissues and normal controls. **(B)** IHC analysis of G3BP2, Oct-4 and Nanog protein expressions in CRC tissues and normal controls. **(C, D)** IF analysis of CD133 and CD44 protein expressions CRC tissues and normal controls. **(E)** Western blot analysis of G3BP2, Oct-4, Nanog, CD133 and CD44 protein expressions in CRC tissues and normal controls. *P<0.05; ***P<0.001.

**
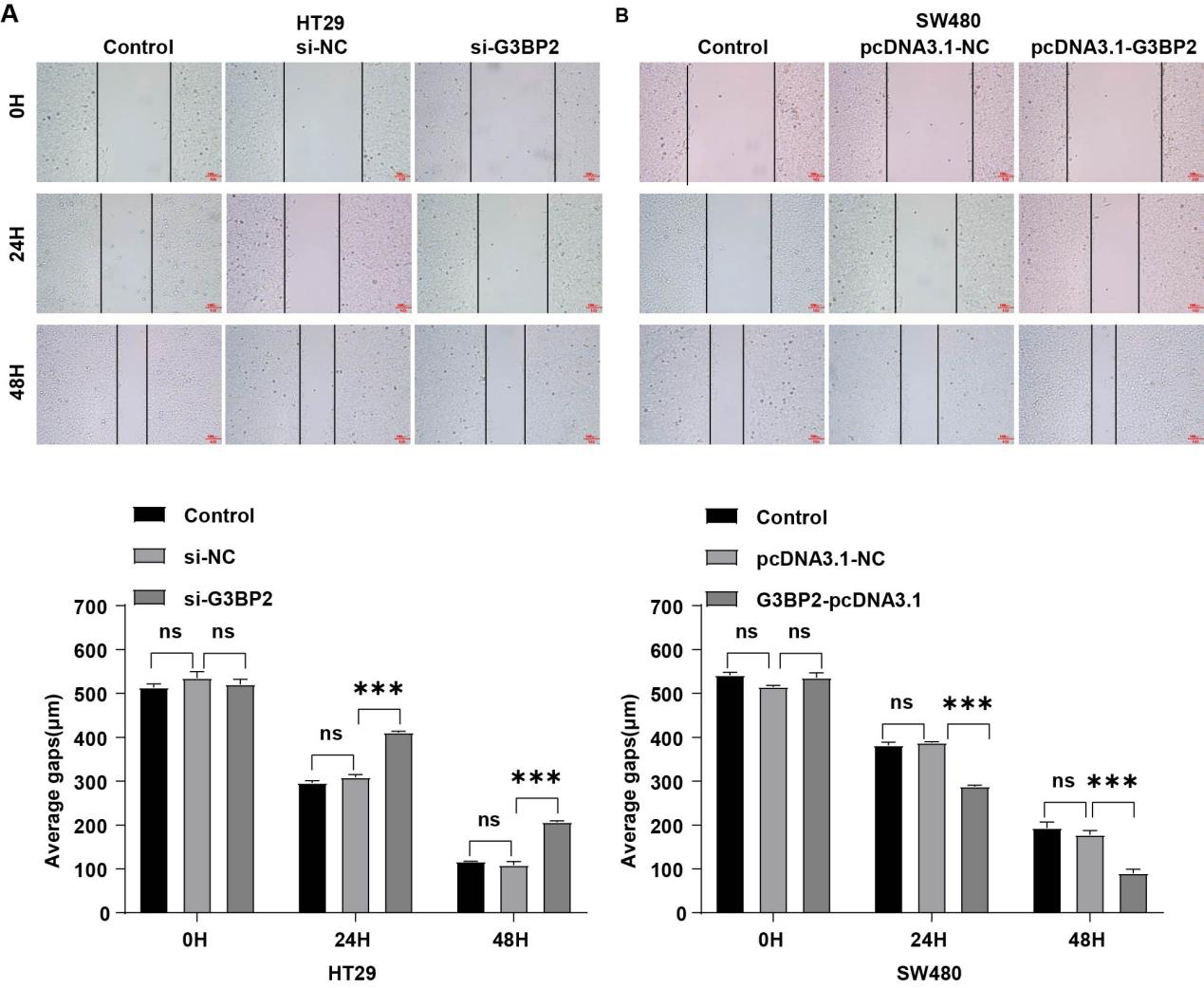
Figure S3 G3BP2 overexpression enhanced CRC cell migration.** HT29 cells were transfected with si-G3BP2 and SW480 cells were transfected with pcDNA3.1-G3BP2. **(A, B)** Wound healing assay were performed to assess cell migration. ***P<0.001.
